# Fabrication and evaluation of bioresorbable scaffolds for interventional cardiology application with sufficient drug release 

**DOI:** 10.22038/IJBMS.2022.62759.13889

**Published:** 2022-03

**Authors:** Asghar Sadeghabadi, Seyed Khatiboleslam Sadrnezhaad, Azadeh Asefnejad, Nahid Hassanzadeh Nemati

**Affiliations:** 1 Department of Biomedical Engineering, Science and Research Branch, Islamic Azad University, Tehran, Iran; 2 Department of Materials Science and Engineering, Sharif University of Technology, Tehran, Iran

**Keywords:** Coronary stent, Degradation, Diffusion, Drug delivery, Nano-hybrid

## Abstract

**Objective(s):**

Bioresorbable scaffolds have been advocated as the new generation in interventional cardiology because they could provide temporary scaffolds and then disappear with resorption. Although, the available stents in clinical trials exhibited biosafety, efficacy, no death, and no apparent thrombosis, Mg-substrate degradation on drug release has not been investigated.

**Materials and Methods:**

Therefore, more research has been needed to legitimize the replacement of current stents with Mg-based stents. UV-Vis spectrophotometer, scanning electron microscope (SEM), X-ray diffraction (XRD), pH measurement, H₂ evolution, and corrosion tests determined the change in hybrid properties and drug release rate.

**Results:**

The effect of Mg degradation on drug release from poly-L-lactide (PLLA) specimen was much higher than that of the L605/PLLA sample. Hydrogen evolution caused by magnesium degradation compelled everolimus out without significant PLLA decomposition during the first 100 days, while formation of Mg(OH)_2_ caused the PLLA to deform and crack.

**Conclusion:**

A combined mechanism of lattice/hole diffusion-dissolution governed the release of everolimus with the activation energies of 5.409 kJ/mol and 4.936 kJ/mol for the first 24 hr and diffusion coefficients 6.06×10^-10^ and 3.64×10^-11^cm^2^/s for the 50^th^ to 100^th^ days. Prolonged suppression of hyperplasia within the smooth muscle cells by hybrid stent insertion could bring about the cessation of restenosis.

## Introduction

One-third of all deaths in industrialized and stressful countries are due to coronary artery atherosclerosis (1). Currently, Drug-eluting stents (DES) loaded with anti-proliferative agents are used in Percutaneous Coronary Intervention (PCI) to prevent restenosis (2). However, DES has its problems. These implants in the coronary artery will limit the normal blood flow and cause an inflammatory reaction of neointima, leading to an immunological reaction and hyperplasia, and in-stent restenosis (3). Also, implanted DES will permanently interfere with future surgical revascularization. The anti-proliferative agent of DES suppresses the vascular smooth muscle cells hyperplasia and results in late in-stent thrombosis (4).

Bioresorbable stents have been innovated for overcoming these problems. These types of scaffolds provide a temporary framework and then disappear into the bloodstream. They eliminate thereby the potential side effects like late blockage. They will also reduce neointimal hyperplasia after stent implantation, resulting in elimination of late in-stent restenosis (5, 6). Due to the concerns about the PLLA stents, magnesium alloy remains one of the most promising bioabsorbable technologies field (7). Magnesium has more electronegativity than other metals used for implants, so it has anti-thrombogenic properties in the body. Magnesium-based substrates are easily corroded and degraded in aqueous media to produce hydrogen gas and magnesium hydroxide. Magnesium hydroxide is insoluble in water but is not stable in the presence of anions or acidic situations. Therefore, decomposition of magnesium in the substrate inevitably changes the environmental conditions (magnesium ion concentration, pH, the partial pressure of hydrogen, and other physicochemical properties). The feasibility and safety of degradable magnesium alloy implants have been determined in *in vivo* tests and clinical trials. Magnesium stent will be able to maintain its mechanical properties for the desired period included in the arterial vessel’s design. 

Also, this medical device has exemplary biocompatibility, and its use has been reported without in-stent thrombosis and mortality (8, 9). The WE magnesium alloy series forms the leading platform of the bioabsorbable stents. Excellent properties such as slow degradation in aqueous solutions and good electrochemical properties in addition to excellent mechanical properties make WE43 alloy a suitable choice for use in cardiovascular implants (10, 11). It inhibits smooth muscle cell proliferation and has anti-proliferative and immunosuppressive properties (12-25). Therefore, everolimus was selected as by-stent carried-drug of this research. L605 is an inactive stent alloy, resistant to corrosion with good mechanical strength. Orsiro^TM^ (Biotronik AG, Bülach, Switzerland) is an L605 stent. It has a base Co-Cr alloy and is coated with an amorphous silicon carbide layer and PLLA as a carrier for sirolimus. Orsiro^TM^ holds the record for the lowest strut cross-section (Ultrathin stent) and causes the least damage to the vessel wall’s endothelium layer (24-31). 

The major target of this study is understanding of effect of Mg-substrate corrosion on drug release from nanohybrid stent for improvement of drug release kinetics. In this research, the solvent casting method produced a hybrid system made of WE43 Mg alloy substrate coated with PLLA, which carried the everolimus drug, and was compared with a permanent control specimen (L605 alloy). Calculation of drug release parameters for bioabsorbable (WE43 based) and Non-bioabsorbable (L605 based) stents revealed both the dominant drug release mechanism and the magnesium degradation effect. All coatings had a similar composition, thickness, and drug content. This study aimed to investigate the effect of magnesium degradation on drug release from a nano-hybrid cardiac stent made of magnesium alloy, which was systematically designed and performed by several experiments. Drug release from two samples representing advanced stents non-absorbable with L-605 base and absorbable with WE43 base was measured under different conditions and times and the results were analyzed.

## Materials and Methods

***Materials preparation and characterization***

**Figure 1:**
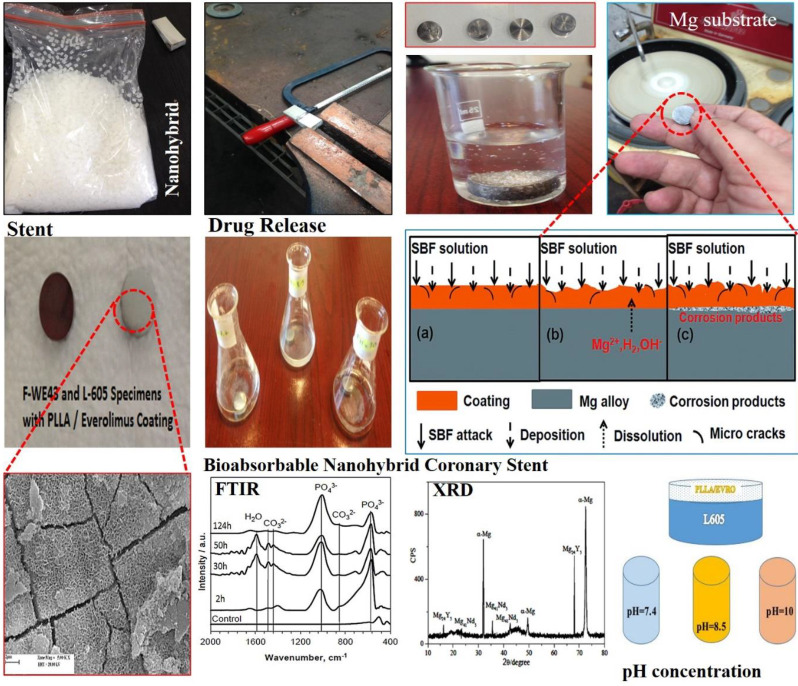
Schematic representation of the polymer-drug nanohybrid coating prepared with WE43 and L605 substrates

Biodegradable magnesium (WE43) alloy with the composition listed in Table 1 was used as the substrate material. Alloy L605 (cobalt alloys) with the detailed composition listed in Table 2 was employed as the control specimen. Poly-L-lactide (PLLA) with an average weight of ~100,000 g/mol (Cas No: 33135-50-1) was purchased from Sigma-Aldrich, USA. Everolimus (>98%, CAS registry No: 159351-69-6). The starting materials of ethanol by Sigma-Aldrich (USA), hydrofluoric acid (40 wt.%), and NaOH were obtained from Merck company as solvent of the product. The specimens were cut from L605 and WE43 alloy with dimensions (Ѳ 14 × 4 mm and a disc shape. Then, the samples were polished in three steps with SiC papers and aluminum oxide to a final level of 2000 grit and cleaned in distilled water, acetone, and ethanol, respectively. Then, the samples were dried with hot air for 2 min at 40 °C. The corrosion resistance of magnesium alloy is low for clinical applications. Therefore, all specimens based on WE43 were treated with hydrofluoric acid (HA) for 10 hr before forming a protective MgF₂ layer with a thickness of approximately 1 μm to slow down the corrosion rate of bare magnesium alloy (WE43). The underside and perimeter of the specimens were carefully covered with waterproof varnish. The PLLA nanohybrid coating was created by the solvent casting method. In this method, the polymer was dissolved in a suitable solvent and the drug was dissolved separately in a suitable solvent. The drug solution was then added to the polymer solution and thoroughly homogenized. To cover the specimens, 0.3 ml of this solution was coated on the surface of the F-WE43. This solution contained 225 g of the drug. Due to the specimens’ surface area, which was equal to 154 mm², the dose of the drug in each specimen was equal to 1.46 µg/mm² (equivalent to standard stent). The samples were then dried in a chemical hood for 36 hr to achieve a similar coating thickness. All coated disc specimens were then transferred to a vacuum chamber for further drying for 50 hr. After that, in order to avoid effect of heat and pressure on the nature of the drug and the polymer, the solvent was evaporated at room temperature, and the polymer coating containing drug nanoparticles formed on the surface of the specimens as shown in Figure 1. For simplicity, acronyms for specimens are listed in Tables 3–5. A grating device by Jean Wirtz (High purity, Germany) was used for specimen grating. Digital electronic scale, pH meter (DENVER INSTRUMENT (USA)), UV/VIS spectrophotometer (Germany) Analytik Jena SPECORD S600 SOFTWARE: winaspect®), Optical Microscope (OLYMPUS BX51 (JAPAN)), SEM (LEO, Model: 1455VP, (Germany)), and Timer (CASIO) were used in the study.

***In vitro EVRO release of F-WE43 and L605 specimens***

This experiment was performed by immersion of the specimens in two separate containers with 10 ml SBF solution, to measure the concentration of the released drug at three temperatures (1 °C, 28 °C, and 65 °C) during the first 24 hr of immersion and secondly to measure the released drug concentration after 100 days after immersion. UV-Vis measurements may support the cumulative everolimus release evaluation. The solution was replaced with the same amount taken to maintain the sink condition. To calculate the percentage of drug released, the amount read by the spectrophotometer (mol/l) was converted to g/ml using the molar mass of the drug, which was 958.244 g/mol, multiplied by 10 (the amount of drug in 10 ml of solution) and was divided by the total amount of drug in the specimen (225 µg) and multiplied by 100.

***pH change during drug release by specimens***

During the release test at each sampling stage after 100-days of experiments, the solution samples’ pH was measured and recorded. F-WE43 sample (without polymer coating) was used separately as a control sample. 

***Effect of pH on drug release***

The effect of media pH was tested by monitoring drug release profiles from the coating on L605 disc specimens in two stages of 24 hr and 100 days. The inactive substrate’s use was to check the pH effect without degradation on the coating’s drug release. 10 ml of SBF solution with three pHs of 7.4, 8.5, and 10 were used. To adjust the required pH of SBF solutions, NaOH solution (0.1 N) was used with an average temperature of 29 °C. From the predetermined time, the solutions were replaced with a fresh solution at the same pH to maintain a constant pH for 24 hr and 100 days until the end of the test process and the collected solution was delivered to the laboratory to measure the release rate. The drug released by the spectrophotometer at wavelength λmax = 289 nm was measured ten times and its percentage was calculated.

***H₂*** ***evolution*** ***test according to ASTM G1-90 standard***

**Figure 2:**
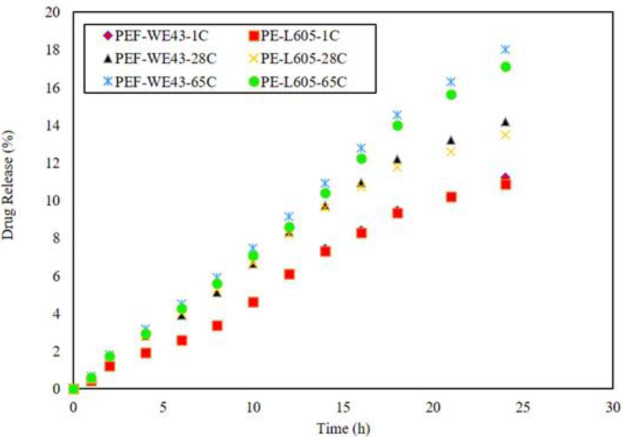
Measurement of hydrogen evolution from the magnesium alloy specimen in SBF solution

The main advantage of the H₂ test was direct measurement. Therefore, the results showed the amount of hydrogen production directly at different stages of Mg degradation. In the early stages of corrosion, a small amount of H₂ (g) was released, making it difficult to collect and measure accurately, in which the solubility of H₂ was a source of error. It depended on temperature and the average area of the specimen in the aqueous solution. As a result, this test had acceptable accuracy for magnesium alloys. Three specimens of F-WE43, PF-WE43, and PEF-WE43 were used and the disc specimens were first immersed separately in a container containing the SBF solution. A glass funnel was placed on top of each specimen to collect the hydrogen that gradually evolved as shown in Figure 2. A scaled portion was placed on the funnel and filled with the solution. According to experiment 1, the desired temperature conditions were provided and the estimated time in terms of the hour, and disc area was about 154 mm². The volume of hydrogen produced and mass loss were measured and recorded at three temperatures of 1 °C, 28 °C, and 65 °C. 

***Everolimus stability***

Degradation of the magnesium alloy substrate causes the environment to become alkaline, which may affect the drug’s effectiveness. Therefore, it was designed as follows for experimental reliability. A 10% solution of the everolimus was used, and its stability was measured in four alkaline, acidic, oxidizing, and natural environments at three temperatures of 1°C, 37 °C, and 60 °C. In this experiment, HCL (2 N) solution, alkaline NaOH (2 N) solution, 20% H₂O₂ solution for oxidizing medium, and SBF solution (pH:7.4) were used to create a neutral environment. At each step, 10 ml of the solution containing the drug was added to 10 ml of the above solutions, and after 30 min, the amount of drug remaining was measured and recorded by a UV-Vis spectrophotometer. 

***XRD analysis***

The X-ray diffraction (XRD) pattern of the WE43 specimen before immersion and after 30 days of immersion was also gained. The XRD was used to characterize the material phase and structure of the component. The crystalline size and physical properties of the tissue were investigated using XRD analysis.

***SEM analysis***

The effect of magnesium degradation on PLLA was investigated by immersing PEF-WE43 and PE-L605 specimens separately into 10 ml of SBF (pH = 7.4) for different durations. The solution was changed regularly in each container. After immersion in SBF for a predetermined time, the specimens were taken out, washed thoroughly with deionized water, and dried in a vacuum chamber. The PLLA coating surface’s morphology on both L605 and WE43 substrates and the WE43 specimen were observed using an optical SEM microscope.

## Results

**Figure 3:**
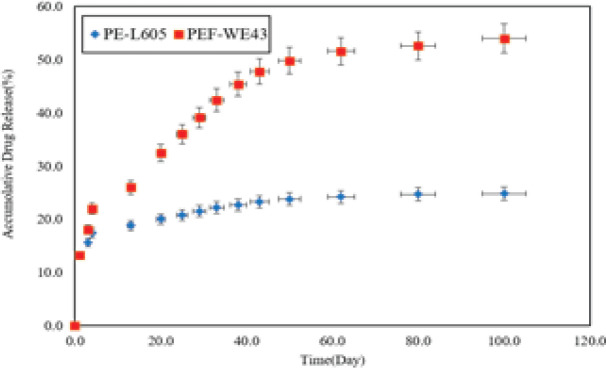
Drug release of PEF-WE43 and PE-L605 at 1, 28, and 65°C in 24 hours

*In vitro*, everolimus release from two specimens at 24 hr was determined at 1°C, 28 °C, and 65 °C, as well as 100 days at room temperature. The best-fit model calculations determined the release mechanism and activation energy of the release processes. Temperature significantly affected the drug release rates and it is obvious at temperatures above 65 °C, separation of the polymer coating from the surface of the metal substrate can occur because the glass transition temperature of PLLA coating is at 63 °C. Higher temperatures accelerated the degradation of magnesium substrate and produced more hydrogen, which affected the drug release according to Figure 3(a). Figure 3(b) indicates the 100-day graph of three distinct phases for both specimens; first, burst release in the first 24 hr, which continued until the fourth day, the second low release of the drug from the third day to the 43rd day, and linear release from 50th to 100th day. The PEF-WE43 specimen showed a significantly higher release rate than the PE-L605 sample. The full PEF-WE43 release was approximately 54%, while that of PE-L605 was only 24.8%. This difference indicates significant effect of Mg substrate corrosion also the difference corresponding to the manufacturer of the stents (32-38).

***pH change of media during drug release***

**Figure 4.**
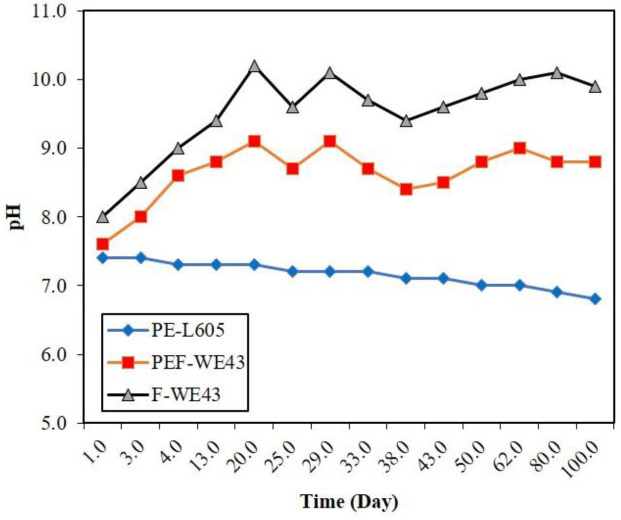
pH media change by PEF-WE43, PE-L605, and F-WE43 specimens during 100 days of immersion in the SBF [according to our previous work]

Changes in the pH media were monitored during the drug release process. A magnesium alloy specimen without polymeric coating was used as a control specimen. The obtained results are shown in Figure 4. For PEF-WE43, the pH has an oscillatory trend: rising from 7.4 to 9.1 in the first 20 days, going down to 8.7 on the 25th day, reaching 9.1 on the 29th day, decreasing to 8.8 on the 50th day, increasing to 9.0 on the 62nd day, and finally reducing to 8.8 on the 100th day. The degradation rates of Mg alloy raised the pH, and then the pH was slightly adjusted by local hydrolysis of PLLA, which resulted in the release of acidic products. In the PE-L605 stent specimen, the pH with a gentle slope decreased slightly, with a shallow slope, and finally reached 6.8 at the end of the 100th day. This pH reduction is attributed to the slow hydrolysis of PLLA. PLLA hydrolysis produced intermediates resulting in decomposition with some acid-base functional groups, such as carboxylic groups, therefore, the pH decreased slightly. In the control sample F-WE43, the pH increased due to production of magnesium hydroxide and reached 10.2 on the 20th day. Then, the downward trend took place and reached 9.4 on the 38th day. In the following days, the trend of changes increased and reached 10.1 on the 80th day. These changes were due to the magnesium’s corrosive nature and formation and dissolution of the protective coating film. Due to the lack of PLLA coating, acidic products did not appear in this specimen, in which no slight decrease in pH was observed.

***Effect of pH on drug release***

**Figure 5:**
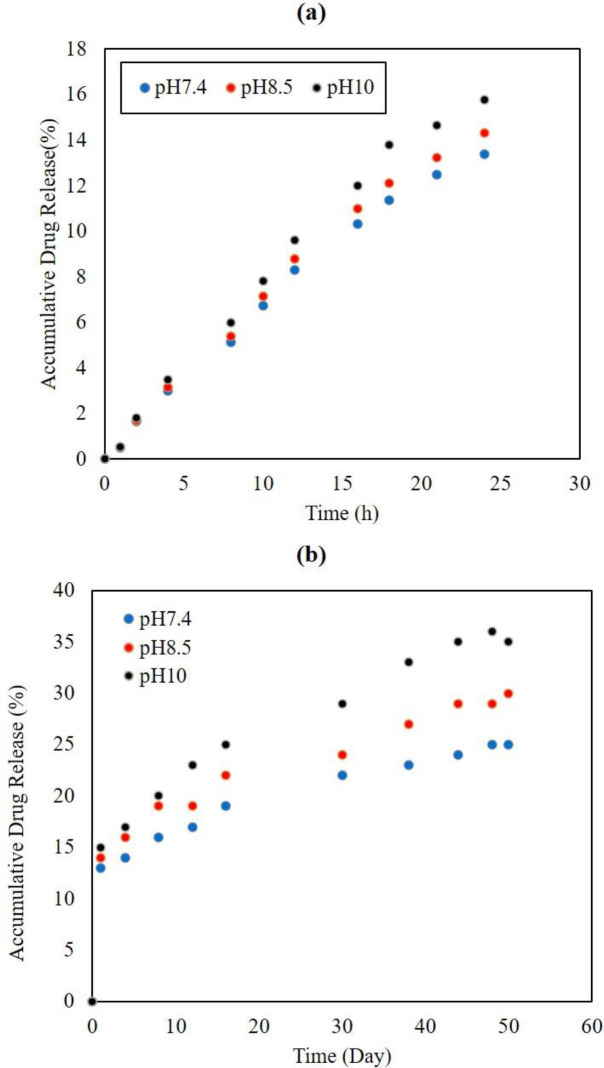
(A) Effect of pH on the everolimus release from PE-L605 control specimen in the first 24 hr of experiment, and (B) Effect of pH on everolimus release from PE-L605 control specimen during 50 days

In the first 24 hr, the amounts of drug release at pHs 7.4, 8.5, and 10 were 13.4%, 14.31%, and 15.78%, respectively as shown in Figure 5(a). The pH change in the tested range did not have much effect on drug release. At the end of the 50th day, the amounts of drug release at pHs 7.4, 8.5, and 10 were increased 24.2%, 30.0%, and 37.0%, respectively (see Figure 5(b)). Prolongation of the SBF specimens’ immersion time increased the pH effect on drug release, which was attributed to the retention time of the PLLA. At pHs higher than 9, this effect was more significant. However, in general, pH in this range did not substantially affect drug release from the PE-L605 specimen.

***H₂ evolution test***

Interaction between PLLA and the substrate WE43 and the hydrogen gas produced by the degradation process often affects and weakens the protective effect of the coating. Relatively high corrosion rates of magnesium alloys are observed in the *in vivo* and SBF solution. This high rate of corrosion is attributed to magnesium levels’ inability to passivate naturally in chloride-containing aqueous solutions at physiological pH levels and the effect of other invasive ions such as phosphates, sulfates, and carbonates. Magnesium alloys are susceptible to corrosion due to their galvanic activity among their significant constituents. These compounds include the initial phase α, the α-reactive phase, the β phase, and the alloying elements. Corrosion attacks often involve galvanic coupling between the anodic and cathodic regions, resulting in preferential dissolution (anodic) of the Mg matrix (phase α), leading to attenuation and secondary phase particles and the cathodic reaction with water. In magnesium corrosion, small holes form on the surface and gradually expand on the surface, but due to the natural and inherent limitation of local corrosion, magnesium does not tend to form deep cavities. The general formula for Mg corrosion shows that the decomposition of a magnesium atom in aqueous media produces a hydrogen molecule. Therefore, the results of the cathodic reaction mainly led to the gradual production of hydrogen. The measured volume of hydrogen gas is equivalent to the mass loss of magnesium. The immersion test is performed according to the following equation:

$\mathrm{Mg}+{2H}_{2}o\to {\mathrm{Mg}}^{+2}+2{\left(\mathrm{OH}\right)}^{-}+|H_{2(g)}↑$


 (1)

Thus, one mole of consumed Mg (24.31 g) produces the equivalent of one mole of H₂ gas (22.4L). H₂ production rate (*V*_H_ ml/cm²/day) is related to the rate of Mg weight loss (Δ*w*) (25, 28):

$∆W=∆W=1\cdot 085V_{H}$


(2)

**Figure 6:**
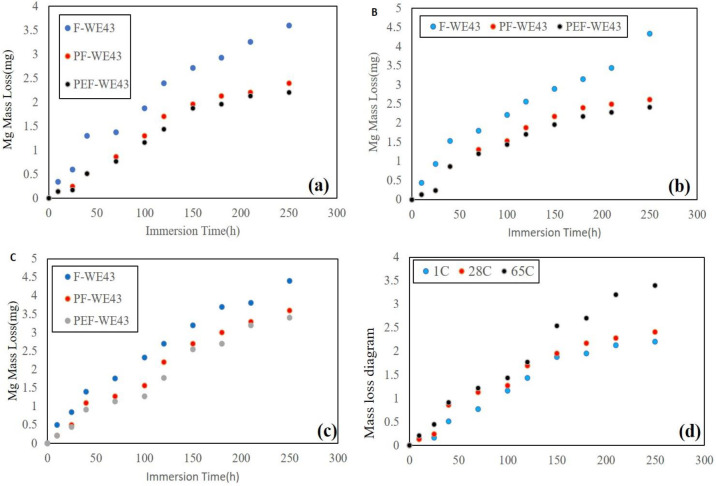
(A) Mass loss diagram of specimens at 1 °C, (B) at 28 °C, (C) at 65 °C, and (D) Mass loss diagram of PEF-WE43 specimen at 1, 28, and 65 ºC

Therefore, from the recorded results and the above formula, the amount of mass loss in the specimens is calculated. The results are shown in the diagrams in Figure 6. As can be seen, the uncoated specimen has a higher corrosion rate than the two coated specimens. 

***Effect of magnesium degradation on EVRO stability***

**Figure 7:**
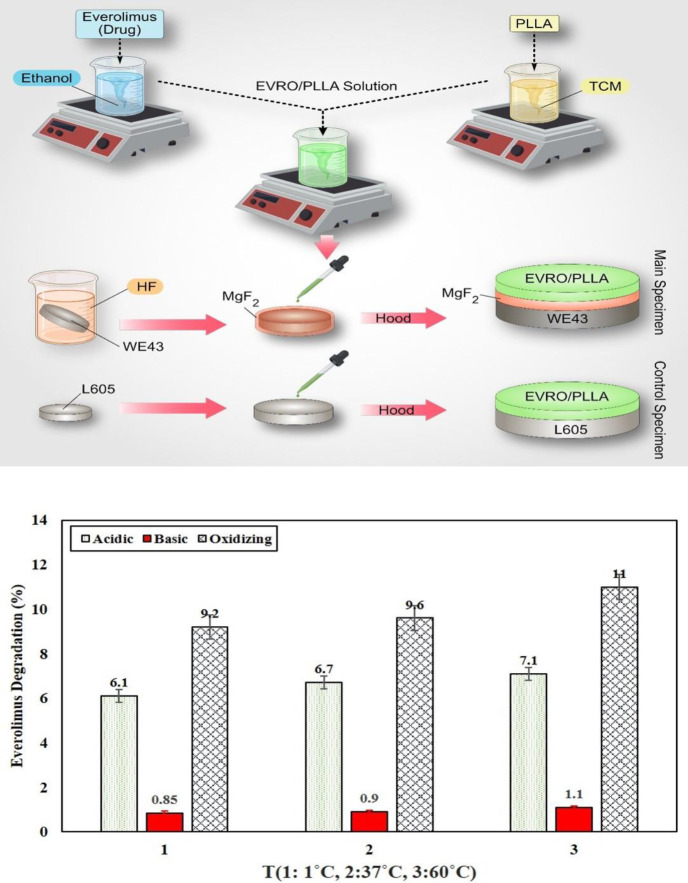
Stability chart of everolimus in different media at different temperatures

Based on the experiments’ results, it is observed that the degradation of everolimus in an alkaline environment in the most severe case (pH=14, temperature 60 °C) is equal to 1.1%. The pH change at its highest by the PEF-WE43 specimen was 9.6. Therefore, the amount of drug destruction in this environment will be meager. It should be noted that in SBF medium (pH:7.4) the percentage of drug degradation was zero. The results are shown in the diagrams in Figure 7.

***XRD analysis***

**Figure 8:**
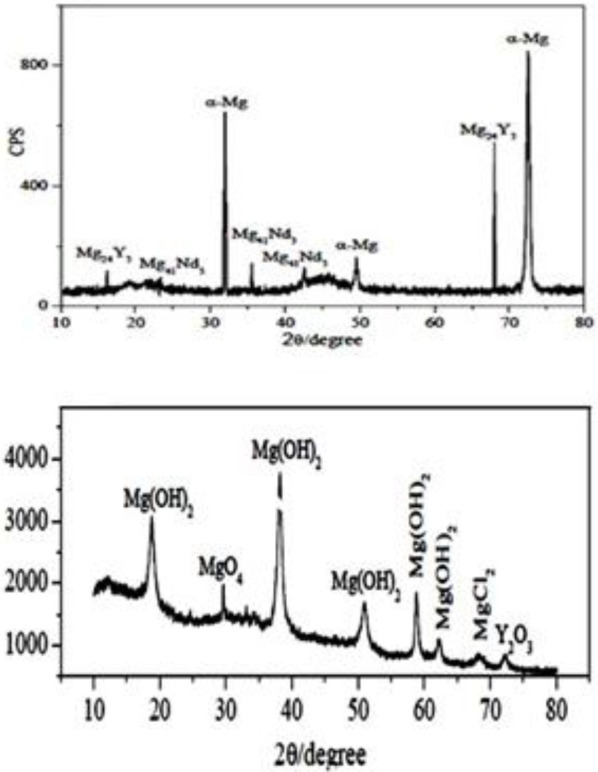
(A) XRD pattern of WE43 alloy before immersion, and (B) XRD analysis of WE43 alloy corrosion product immersed in SBF after 30 days

The XRD pattern for WE43 alloy is shown in Figure 8A. This pattern shows the presence of α-Mg,, and in the produced layer. This layer also consists of (RE) and Y-rich phase. These phases have positive potentials relative to the magnesium matrix, so they act as sites for hydrogen production by the cathodic half-reaction of the corrosion. In addition, rare earth elements in the WE43 alloy are involved in the removal of impurities (such as Fe, CL, S, O, and H). This phenomenon can change impurities in the alloy from the state of the material in solution to intermetallic compounds. This phenomenon helps increase corrosion resistance. During the WE43-SBF immersion test, severe corrosion occurred with large cavities and cracks. As the immersion time increased, all alloys were severely degraded and the surface of WE43 alloy was covered with a large amount of corrosion products. The surface was severely damaged and a large number of superficial holes were formed. When the test was completed, the corrosion products were collected from the alloy surface by a stainless-steel knife. XRD analysis shown in Figure 8B indicates the corrosion products after 30 days of immersion in SBF. Mg (OH)₂, the main corrosion product, was on the magnesium alloy. In WE43 alloy, MgO₄, MgCl₂, and Y₂O₃ can also be present in the product phase. The oxidized Y bond at the surface of the coating film and the Y-rich region in the matrix can effectively delay the biodegradation of the magnesium alloy (27-33).

***Optical and SEM images***

**Figure 9:**
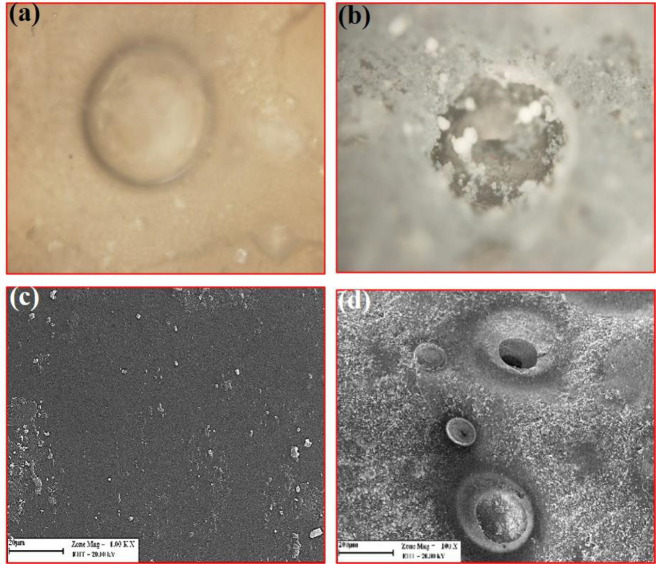
Blisters (A) formed by hydrogen bubbles inside the PLLA due to the magnesium-water reaction under the layer after six days (100x magnification), and (B) partial destruction of the coating after 14 days (100x magnification)

Figure 9-A shows a blister created by H_2_ accumulation inside the PLLA coating after 6 days of immersion into the SBF. The blister grows to reach the size seen in Figure 9-B and then bursts. The burst causes severe local damage to the drug-containing polymer coating, increasing EVRO expulsion. In the SEM images, on the first day after immersion of the specimen (Figure 9-C), a gradual deposition of SBF-soluble salts on the surface was observed. However, the coating was still intact. On the twentieth day of immersion (Figure 9-D), large blisters of various sizes were present on the cover plus sediment. The existence of these blisters was due to penetration and passage of SBF through the polymer coating toward the substrate to cause the magnesium-water reaction for hydrogen and magnesium hydroxide production. Hydrogen gas has a high diffusion rate and evolves to the environment. Due to the semi-crystalline nature, the coating traps some hydrogen, which causes blistering. In other words, the formation of hydrogen bags causes microscale swelling of the coated Mg substrate. The increased surface area of the polymer coating can facilitate penetration according to Fick’s law of diffusion. The magnesium hydroxide produced causes localized scaling, too. Four stages can be considered to explain the everolimus release mechanism such as drug transfer in the PLLA solid phase, pore diffusion in the blistered PLLA layer, drug dissolution at the polymer-SBF interface, and convective everolimus transfer into the bulk liquid away from the interface. From the above separate steps, (1) and (2) are parallel; while (4) consecutively occurs after (3) which happens next to (2) or (1). Assuming a well-stirred solution, the fourth step becomes insignificant. Step (3) is considered a first-order isothermal reaction represented by a dimensionless time in which *X* is the fractional release defined by:

$X=\frac{\mathrm{drug released mass}}{\mathrm{overall loaded drug}}$


 (3)

The overall (1-2) transfer steps are considered by the quadratic term adopted from the well-established shrinking-core unidirectional flat system comprising a first-order reaction in series with internal diffusion (28):

$t^{*}=g_{F_{g}}\left(X\right)+\varphi_{g}^{2}P_{F_{g}}(X)$


 (4)

is the overall dimensionless time of the whole release process, is the dissolution part, and the is the internal diffusion share. The term called Thiele modulus is a cognitive factor for the diffusion term summation with dissolution duration (26, 38-44). Adaptation of test data during the first 24 hr showed that drug dissolution was a control step at all three temperatures. Figure 12 shows the best-fit curves for both PEF-WE43 and PE-L605 samples during the first 24 hr of immersion. The activation energy for the dissolution of everolimus into the SBF was determined from the Arrhenius equation:

$\tau =\tau_{0}e^{\frac{Q}{\mathrm{RT}}}$


(5)

Where *τ* is the total conversion time, *Q* is the activation energy, *R* is the ideal gas constant, and *T* is the absolute temperature. The activation energy for the PEF-WE43-PLLA sample was 5.409 kJ/mol, while for the PE-L605-PLLA sample, it was 4.936 kJ/mol. These values are in agreement with the previously reported drug dissolution activation energies (45-51). Calculated results for 24 hr drug release from the synthesized samples are summarized in Table 5. Matching the drug release data of PEF-WE43 for times between 4 and 43 days shows a mixed control dissolution-diffusion mechanism with the equation. For the period between 50 and 100 days, the data indicates a mere diffusion control for the PEF-WE43 sample. However, the governing mechanism for PE-L605 after day 4 is mere diffusion with relationship constants.

## Discussion

Magmaris^TM^ (Biotronik AG, Bülach, Switzerland) is a bioabsorbable and expandable scaffold with a balloon. The stent backbone is made of WE43 magnesium alloy and is entirely radiolucent. This stent has two tantalum permanent radiopaque markers on the distal and proximal ends (52-59). The backbone surface is completely coated with a 7 µm biodegradable PLLA loaded polymer with sirolimus (BIOlute) similar to Orsiro^TM^ (Biotronik AG, Bülach, Switzerland) platform (13). Magmaris was successively tested in BIOSOLVE II, BIOSOLVE III, and BIOSOLVE IV clinical trials (Table 1), demonstrating biosafety and efficacy with no death and no apparent scaffold thrombosis (14). However, the effect of Mg-substrate degradation on drug release has not been investigated. Therefore, more research has been needed to legitimize the replacement of current stents with magnesium alloys. In the case of the stent platform, one of the most challenging issues for the use of magnesium alloys is the high rate of degradation and local corrosion. Surface treatment with hydrofluoric acid creates an MgF₂ conversion layer to increase the durability and biocompatibility on the magnesium alloy’s surface and has corrosion protection properties in magnesium, and has shown a reduction in corrosion rate in these alloys. However, the protection of this layer is temporary (18-19). PLLA is a biodegradable semi-crystalline polymer that is degraded in 3 steps and is metabolized to CO₂ and H₂O (20). For this reason, it is widely used as a drug carrier in drug delivery and scaffolding for tissue engineering, drug-carrier and coatings for stents, and other similar implants (20-24). The side effects of sirolimus have been of concern too (23). Everolimus not only has fewer side effects but also prevents in-stent stenosis (24). Also, it is pharmacokinetically better than the formerly used substances. This drug has wide use as an anti-restenosis agent in DESs, too. From the total conversion times ( inserted in the governing correlations one can obtain the internal diffusion coefficients from equation (6):


(6) $$\tau_{\mathrm{dis}}=\frac{\rho^{L^{2}}}{{\mathrm{bk}}_{\mathrm{dis}}C}$$

Where is density, L is the layer thickness, b is the stoichiometric constant (considered one, here), is the dissolution rate constant, and C is the drug composition of the sample-SBF interface. The lower total conversion time of PEF-WE43 than that of PE-L605 indicates the degradation effect of Mg. The diffusion coefficient of PEF-WE43 is thus higher than PE-L605 due to faster transfer in the blistered layer. The dissolution/diffusion mechanism change from PEF-WE43 to PE-L605 indicates the latter’s slower drug transfer due to the dense/non-blistered PLLA on PE-L605. The dense/non-blistered PE-L605 has a lower diffusion coefficient than PEF-WE43 at longer times. The intensification of the drug release in the PEF-WE43 is due to three factors: alkaline environment, H₂ evolution, and corrosion products. While the evolution of H₂ has a significant consequence in increasing drug diffusion rate, the effect of pH on drug release is low. The inactive specimen (PE-L605) has neither H₂ evolution nor alkaline medium formation. In the coated specimens, direct contact of PLLA with the aqueous solution causes a hydrolysis reaction catalyzed by an acidic or alkaline medium. The hydrolysis leads to pores and cracks in the polymer coating and more intermediate decomposition, mainly oligomers and monomers, which typically have carboxylic groups. The decomposition of the polymer is generally autocatalized by the end groups of carboxylic acid and accumulated in the cavities. It has been found that the pH inside the cavities caused by the erosion of anhydrides is between 4 and 5, while the areas far from the surface and inside the bulk are around 7.4. Even lower pH has been observed in the erosion of polymers. For example, for PLLA and similar polymers, lower pH is observed due to higher solubility and lower PKa compared with poly (anhydride) monomers. Therefore, the higher pH of the solution accelerated the hydrolysis of PLLA in the first step. As a result, due to the production of products resulting from pH reduction, a decrease in polymer coating was observed. In addition, a solution with a pH range of 7.4–10 could neutralize the acidic products of the decomposition in the micro medium and thus may prevent the autocatalysis of the final carboxylic acid groups on the gap (opening) of the polymer chain. A previous study showed that the biodegradation rate of PLLA was flattened in a moderately alkaline medium with pH = 9.24 (zero decomposition) while the polymer was decomposed in media at pH = 5.0. Based on previous findings, it can be inferred that PLLA hydrolysis in a play environment with pH<9.7 may not progress (9,11). From the amount of hydrogen produced from the three specimens, it is clear that the production of hydrogen in the uncoated specimen was significantly higher than in the two coated specimens. This difference indicates that more magnesium has reacted with SBF, indicating greater exposure to the solution. It is observed that the uncoated specimen lost a higher mass than the two coated specimens. Nevertheless, the amount of mass lost in the two specimens is close to each other. Therefore, it is clear that the use of PLLA coating can control the corrosion rate of the substrate and hydrogen gas production. MgF₂ is a porous conversion layer. PLLA solvent casting film is dense and attaches to the substrate integrated. PLLA is penetrated into the porous MgF₂ forming an integrated polymer/ceramic composite coating. PLLA facilitates the improvement in adhesion between the coating and substrate compared with the MgF₂ layer because of its flexibility and higher contact zone. This composite coating will prolong the absorption of the implant in the body. Of course, this operation needs to consider many parameters such as coating thickness, type of coating technique, polymer concentration, polymer crystallinity, polymer purity, and polymer-drug homogeneity. The experiments showed that the loaded drug had little effect on substrate corrosion and hydrogen production control. The PLLA served as a temporary absorbable protective coating and a suitable drug carrier for a bioabsorbable stent. In addition to the role of slowing down the corrosion rate and the drug carrier, PLLA also plays the role of maintaining the corrosion products under the layer. This polymer is hydrolyzed in 24 months, but the magnesium alloy decomposes in 12 months. Therefore, the technique of coating magnesium stents with PLLA is used for this purpose so that metal parts caused by corrosion are not left in the body and do not enter the bloodstream. In this way, blood fluids penetrate through the polymer coating and reach the base magnesium and react with it and decompose Mg. After the metallic magnesium alloy substrate is wholly decomposed and turned into corrosion products, the hydrolysis of PLLA is completed, and after the metal part, the polymer coating begins to degrade. Therefore, because the polymer coating is absorbed one year after the metal part, it can be a Biopack that preserves corrosion products. Figures 11 and 12 represent the effect of WE43 degradation on the everolimus release mechanism. The SEM micrograph of the WE43-PLLA specimen immersed in SBF of coating surface after 40 days of immersion is presented in Figure 11. 

**Table 1:** Chemical composition of WE43 used for this research

**Magnesium**	**Zirconium**	**Rare earths**	**Yttrium**
Balance	0.4% min	2.4-4.4%	3.7-4.3%

**Table 2:** Chemical composition of L605 used for this research

**P**	**S**	**C**	**Si**	**Mn**	**Fe**	**Ni**	**W**	**Cr**	**Co**
Max 0.04	Max 0.03	0.1	Max 0.4	1.5	Max 3	10	15	20	Balance

**Table 3.** Best-fit results for 24 hr drug release from the synthesized samples

Q (J/mol)		τ(h)		Dissolution correlation		Temperature (°C)
**PE-L605**	**PEF-** **WE43**	**PE-L605**	**PEF-** **WE43**	**PE-L605**	**PEF-** **WE43**	
4936	5409	204.08	204.08	X=0.0049t R²=0.9881	X=0.0049t R²=0.9895	1
		158.73	153.84	X=0.0065t R²=0.9874	X=0.0063t R²=0.9804	28
		135.14	129.87	X=0.0074t R²=0.9965	X=0.0077t R²=0.9974	65

**Table 4:** Best-fit kinetic data for drug release from the synthesized samples

**Determination coefficient (R²)**	**Governing correlation**	**Time range** **(day)**	**Sample**
0.9952	$\frac{t}{136.99}=X+0.1X^{2}$	4≤t≤43	PEF-WE43
0.9773	$\frac{t}{1000}=X^{2}-0.1979$	50≤t≤100	
0.9971	$\frac{t}{1666.67}=X^{2}-0.0275$	4≤t≤43	PE-L605
0.8537	$\frac{t}{1666.67}=X^{2}-0.0542$	50≤t≤100	

**Table 5:** Calculated results of drug release kinetics

**Specimen**	Period (days)	$\tau_{\mathrm{dis}}$ (day)	D (cm²/s)
**PEF-WE43**	50-100	1000	$6.06\times 10^{-10}$
**PE-L605**	50-100	16667	3$\mathrm{.64}\times 10^{-11}$

PLA: poly-L-lactide

**Figure 10:**
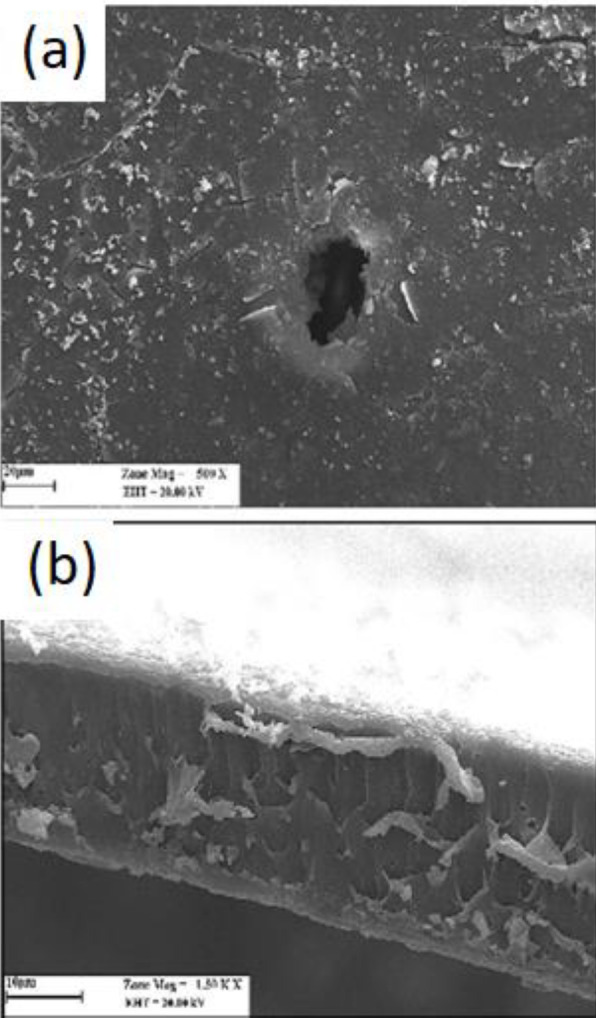
SEM micrograph of the WE43-PLLA specimen immersed in SBF for: (A) 30 days, (B) cross section 30 days

PLA: poly-L-lactide

**Figure 11:**
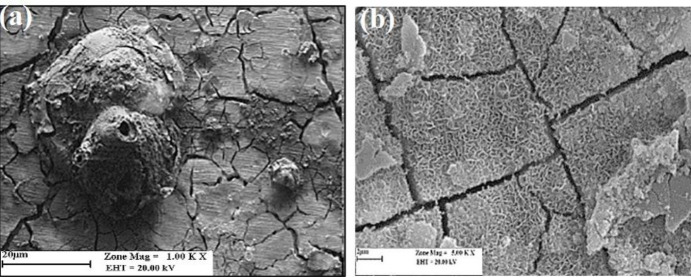
SEM of the corroded surface of WE43 magnesium alloy in SBF (30 hr) and the products of WE43 corrosion in SBF after removal of PLLA coating (15 days)

PLA: poly-L-lactide

**Figure 12:**
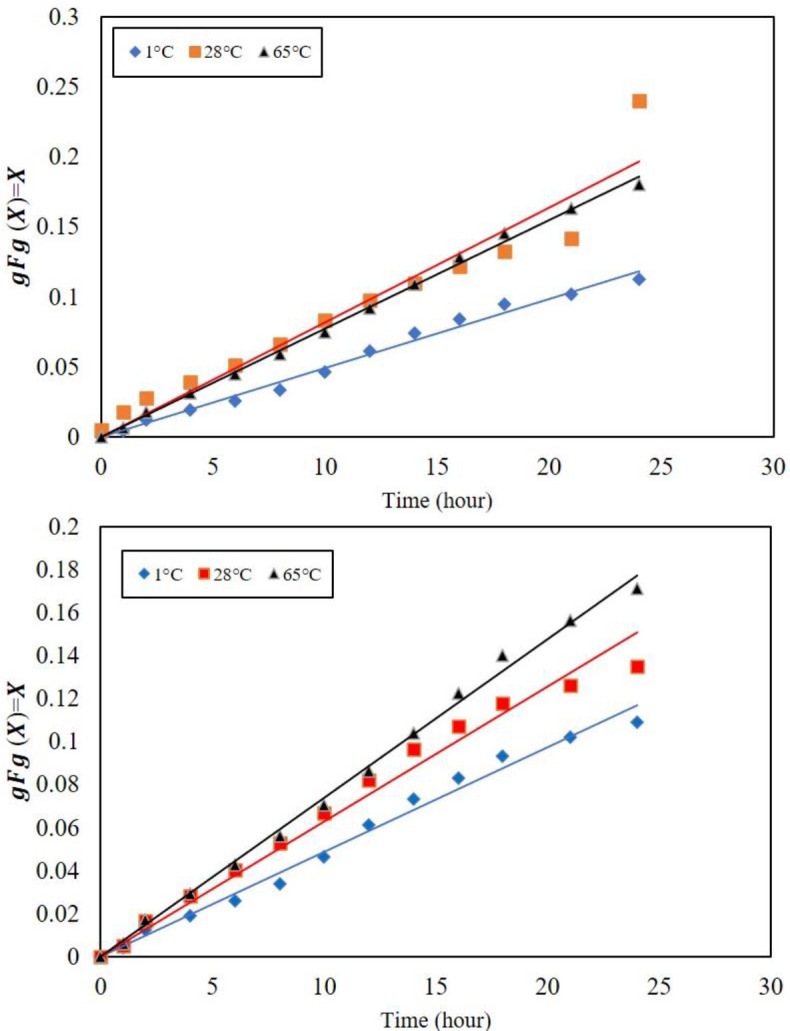
Comparison of experimental release data (geometric shapes) with the model results (lines) for (a) PEF-WE43 and (b) PE-L605 samples during the first 24 hr of immersion

## Conclusion

The everolimus release kinetics of biodegradable WE43 alloy-based specimen were studied in the SBF system in comparison with the passive L605 specimen. The following conclusions were obtained from the investigation. *In vitro* drug release kinetics can be increased by gradual production of hydrogen and corrosion products formed during magnesium-based substrate degradation. Hydrogen gas has a high transfer rate; therefore, it helps the drug release speed. Most magnesium corrosion products have a sharp-edge special shape that cause physical damage to the PLLA coating and furtherance of the everolimus release rate from the F-WE43 specimen. The effect of the weak alkaline pH was negligible on the everolimus release speed. Due to the greater release of the drug in the magnesium alloy specimen loaded with everolimus, it is predicted that the magnesium base stent will be able to prevent smooth muscle cell proliferation (SMCs) and restenosis for a longer period of time compared with non-active substrates drug-loaded (L605). The creation of an alkaline environment due to the destruction of the magnesium substrate may affect the effectiveness of the drug. This effect is, however, not severe. In the first 24 hr, the prevailing release mechanism is dissolution with an activation energy of 5.409 kJ/mol for the coated WE43 specimen and 4.936 kJ/mol for the coated L605 alloy. By increasing the time, the release mechanism tends gradually to convert to internal diffusion due to the difficulties arising from lowering the drug composition in the PLLA layer. The everolimus diffusion coefficient is for the Mg-based sample and for the Co-Cr-based sample during the period of 50 to 100 days from the release start. In the Mg-based sample, the dominant release mechanism is dissolution until the end of the fourth day; from the fourth day to day 43 mixed (dissolution + diffusion) and then until the end of the 100-day period, it is mere diffusion. For Co-Cr based sample, the mechanism during the first day is dissolution, and then until the end of day 100, it is merely diffusion with a lower coefficient. Many factors can affect PLLA erosion. At local pH > 10, damage occurs by H₂ plus Mg(OH)_2_. These factors increase the rate of PLLA degradation and drug diffusion. Intermediate products of the substrate react with the coating and accelerate PLLA degradation. In addition, scaly corrosion products cause physical damage to the PLLA, which affects the release rate. It is noted that this study was performed mainly in SBF solution, the composition of which is different from a real dynamic biofluid system. Many research centers and biomedical engineering companies are currently working on bioabsorbable stents. These activities include the development of more biocompatible materials, ultrathin stents, nanomedicine usage, and better scaffold design to overcome existing limitations. It is believed that nanohybrid bioabsorbable stents based on magnesium alloy are the devices most associated with innovation in coronary interventions and may ultimately lead to long-term clinical benefits and ultimate success.

## Authors’ Contributions

AS Fabricated the coronary stent. SKS, AA, and NHN were his supervisor, co-supervisor, and advisor, respectively.

## Conflicts of Interest

None.
